# Homozygous mutation in *HSPB1* causing distal vacuolar myopathy and motor neuropathy

**DOI:** 10.1212/NXG.0000000000000168

**Published:** 2017-07-06

**Authors:** Enrico Bugiardini, Alexander M. Rossor, David S. Lynch, Michael Swash, Alan M. Pittman, Julian C. Blake, Michael G. Hanna, Henry Houlden, Janice L. Holton, Mary M. Reilly, Emma Matthews

**Affiliations:** From the MRC Centre for Neuromuscular Diseases (E.B., A.M.R., J.C.B., M.G.H., J.L.H., M.M.R., E.M.), UCL Institute of Neurology and National Hospital for Neurology and Neurosurgery; Department of Molecular Neuroscience (D.S.L., A.M.P., M.G.H., H.H., J.L.H.), and Division of Neuropathology (J.L.H.), UCL Institute of Neurology, London; Department of Neurology (M.S.), The Royal London Hospital; and Department of Clinical Neurophysiology (J.C.B.), Norfolk and Norwich University Hospital, UK.

## Case report.

A 57-year-old woman, born to parents of Gujarati Indian descent (figure, A), presented at age 19 with pain and stiffness in her calves and a tendency to trip. In her 20s, a formal neurologic examination demonstrated predominantly distal lower limb weakness and normal upper limb muscle strength.^1^ Motor and sensory nerve conduction studies were normal with the exception that no motor response was elicited from the extensor digitorum brevis. Fibrillations and polyphasic action potentials were present on EMG. The creatine kinase (CK) level was 1,452 IU/L. A quadriceps muscle biopsy was performed at age 27 from which images were available in the records.^1^ At that time, muscle fiber diameters were large ranging from 50 to 80 μm. Many of the fibers contained single or multiple unrimmed vacuoles that appeared empty in the modified Gomori trichrome preparation (figure, B.a). There was no increase in endomysial connective tissue or evidence of inflammation, necrosis, or regeneration. There was no evidence of glycogen, increased lipid, or acid phosphatase staining in the vacuoles (figure, B.b). The ATPase at pH 9.5 demonstrated that most of the vacuolated fibers were of type 2, and electron microscopy showed electron-dense material within vacuoles (figure, B.c and d).^1^ The overall appearances were those of a vacuolar myopathy without any features suggesting neurogenic change, and she was diagnosed with a distal myopathy.^1^

**Figure:**
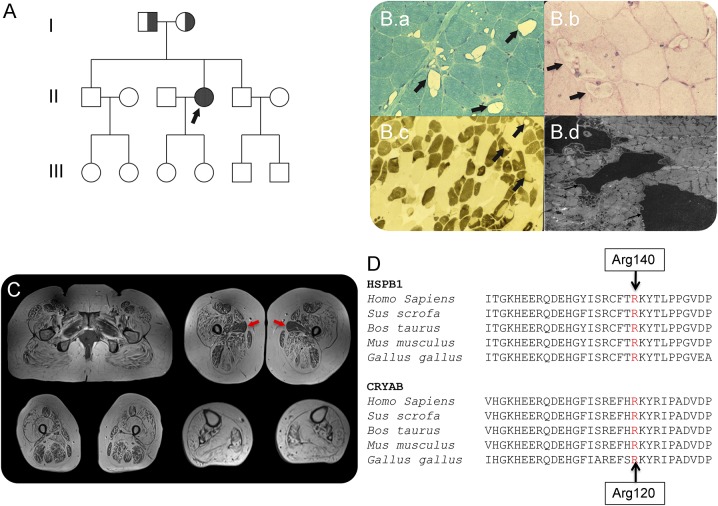
Clinical-pathologic features of patient homozygous for the *HSPB1* p.Arg140Gly mutation and protein conservation between species (A) Family pedigree: an arrow indicates the proband; half-filled indicates distal weakness in parents who were heterozygous for p.Arg140Gly mutation. (B) Biopsy of the quadriceps muscle performed at age 27; (B.a) modified Gomori trichrome staining shows variation in fiber diameter and prominent vacuoles within many muscle fibers, arrows; (B.b) Periodic acid–Schiff preparation showed no evidence of glycogen accumulation within vacuoles (arrows); (B.c) ATPase pH 9.5 demonstrates that vacuoles are predominantly in darkly stained type 2 fibers, arrows; and (B.d) ultrastructural examination of the muscle revealed electron-dense material within vacuoles (arrows). (C) Muscle MRI demonstrating severe widespread fatty infiltration of pelvic, thigh, and calf muscles with relative sparing of the adductor longus (red arrows). (D) Conservation of HSPB1 and HSPB5 (CRYAB) amino acid sequence between species. The Arg140 residue in HSPB1 is well conserved and corresponds to the position of Arg120 in the *CRYAB* gene.

She re-presented at age 57 following the development of slowly progressive severe upper and lower limb weaknesses. On examination at age 57, there was evidence of distal more than proximal upper limb weakness affecting wrist extension (Medical Research Council [MRC] grade 4+/5), finger extension (4 + 5), first dorsal interossei (1/5), abductor pollicis brevis (3/5 right and 4/5 left), and abductor digiti minimi (3/5 right and 4/5 left). In the lower limbs, she had severe proximal weakness (grade 2/3) with no movement at the ankles. Sensory modalities were preserved except for reduced vibration sense at the ankles. She was areflexic. Her CK level was 404 IU/L. Neurophysiologic studies suggested an axonal motor neuropathy. Sensory nerve action potentials were, nevertheless, at the lower limit of normal for amplitude in the lower limbs (right sural 6 μV, right superficial peroneal 7 μV, and normal range >5 μV), and distal lower limb motor responses were absent. Needle EMG showed prominent chronic neurogenic changes with large motor units recruiting in reduced numbers but at increased firing rates to a reduced interference pattern. This EMG pattern was most pronounced distally but evident proximally in the upper and lower limbs. No low amplitude or brief polyphasic motor units were seen on any occasion at re-presentation. Muscle MRI was performed, showing widespread severe muscle fatty replacement (figure, C).

There was no relevant family history. Her mother has diabetes and her father a right above knee amputation for peripheral vascular disease. Neither had neurologic complaints. However, clinical examination of both in their 80s revealed mild distal weakness (MRC grade 4/5) in the upper and lower limbs with areflexia. Pinprick sensation was reduced to the mid-forearm and foot in her mother. Her father had reduced vibration sense to the left ankle. Neurophysiologic testing was not possible in either.

Targeted exome sequencing of the proband's DNA using the Agilent Focused Exome kit identified a homozygous variant (c.418C>G, p.Arg140Gly) in *HSPB1*, which was confirmed by Sanger sequencing. Both parents were heterozygous. We have reported this variant previously in heterozygous form in individuals from 5 Indian Gujarati families with distal motor neuropathy.^2^

## Discussion.

Our patient presented at age 19 with clinical and biopsy features consistent with a distal myopathy. Prominent vacuoles in type 2 fibers contained granular, electron-dense material (figure, B) that was interpreted to represent the product of myofibrillar degeneration.^1^ At presentation, neurophysiologic studies did show fibrillations and polyphasic action potentials. However, nerve conduction studies were normal with the exception of 1 absent motor nerve response, and overall, the clinical image was felt to represent a distal myopathy at this time. Subsequent neurophysiologic studies performed at age 57 following progressive limb weakness revealed an axonal motor neuropathy. Although chronic end-stage myopathy may have neurophysiologic features that can appear neurogenic, in this case, even the less affected proximal limb muscles failed to demonstrate any myopathic motor units or myopathic recruitment.

HSPB1 is a small heat-shock protein highly expressed in striated muscle with an important role in maintaining myofibrillar structure during stress conditions.^3^

Mutations in *HSPB1*, *HSPB3*, and *HSPB8* are classically associated with motor neuropathy.^4^ *HSPB5* (*CRYAB*) has been associated with a wide spectrum of clinical manifestations including desmin-related myofibrillar myopathy. The protein position of the Arg140Gly *HSPB1* mutation in our case corresponds to the Arg120 *HSPB5* residue mutated in this myopathy (figure, D).^4^ Heterozygous mutations in *HSPB8* have recently been reported causing neuropathy and distal myopathy with rimmed vacuoles and fibrillar aggregates in 2 families.^5^ Subsequently, distal myopathy and neuronopathy have been attributed to an *HSPB1* mutation in 1 family.^6^ We describe a patient with a homozygous *HSPB1* mutation also presenting with a distal vacuolar myopathy, motor neuropathy, and minimal sensory involvement, supporting the association of *HSPB1* mutations with this phenotype. This expands the genetic testing indicated in distal vacuolar myopathy.
